# Decreased Temporal Sensorimotor Adaptation Due to Perturbation-Induced Measurement Noise

**DOI:** 10.3389/fnhum.2019.00046

**Published:** 2019-02-14

**Authors:** Elisabeth B. Knelange, Joan López-Moliner

**Affiliations:** Vision and Control of Action Group, Department of Cognition, Development and Psychology of Education, Institut de Neurociències, Universitat de Barcelona, Barcelona, Spain

**Keywords:** temporal, adaptation, Kalman filter, delay, uncertainty, noise, multisensory integration

## Abstract

In daily life, we often need to make accurate and precise movements. However, our movements do not always end up as intended. When we are consistently too late to catch a ball for example, we need to update the predictions of the temporal consequences of our motor commands. These predictions can be improved when the brain evaluates sensory error signals. This is thought to be an optimal process, in which the relative reliabilities of the error signal and the prediction determine how much of an error is updated. Perturbation paradigms are used to identify how the brain learns from errors. Temporal perturbations (delays) between sensory signals impede the multisensory integration of these signals. Adaptation to these perturbations is often incomplete. We propose that the lack of adaptation is caused by an increased measurement noise that accompanies the temporal perturbation. We use a modification of the standard Kalman filter that allows for increases in measurement uncertainty with larger delays, and verify this model with a timing task on a screen. Participants were instructed to press a button when a ball reached a vertical line. Temporal feedback was given visually (unisensory consequence) or visually and auditory (multisensory consequence). The consequence of their button press was delayed incrementally with one ms per trial. Participants learned from their errors and started pressing the button earlier, but did not adapt fully. We found that our model, a Kalman filter with non-stationary measurement variance, could account for this pattern. Measurement variance increased less for the multisensory than the unisensory condition. In addition, we simulated our model's output for other perturbation paradigms and found that it could also account for fast de-adaptation. Our paper highlights the importance of evaluating changes in measurement noise when interpreting the results motor learning tasks that include perturbation paradigms.

## 1. Introduction

Successful sports performance often depends on the ability to make accurate and precise movements in time and space. Yet not all of the movements we make turn out as intended. Our muscles can fatigue, and the world around us changes constantly. Generally, we can correct errors online, however in order to do so, we rely on sensory feedback that is processed by our brain with a delay of around 150 ms for visuomotor tasks (Miall et al., 1993). In order to compensate for these delays, our brain predicts the consequences of our motor commands with a forward model (Jordan and Rumelhart, 1992; Wolpert and Miall, 1996). This internal model predicts the sensory feedback it will receive when a motor command is sent. The difference between the predicted sensory feedback and the actual sensory feedback is called the prediction error (Jordan and Rumelhart, 1992). Error-based learning occurs when we evaluate the prediction error to update the forward model (Wei and Körding, 2009). The process of updating the forward model is called adaptation (Huang et al., 2011). Through adaptation, our predictions of future movements can become more accurate.

Unfortunately, there are many factors unrelated to the accuracy of our motor command that can influence its consequence (He et al., 2016). Trial-to-trial variability in these consequences might stem from neural sources like sensory noise, cellular noise, and motor noise (Harris and Wolpert, 1998; Jones et al., 2002; van Beers et al., 2004; Churchland et al., 2006a,b; Faisal et al., 2008; van Beers, 2009). Note that not all these types of noise are necessarily disadvantageous. Neural noise in the form of stochastic resonance can benefit detection of inputs that otherwise would remain sub-threshold (Faisal et al., 2008; van der Groen et al., 2018). Other sources of noise are inaccurate estimates of the task requirements (Osborne et al., 2005), and disturbances from the outside world (Tan et al., 2016). These different types of noise give the brain the complex task to evaluate which part of an error stems from noise, and which part stems from inaccurate predictions (Wei and Körding, 2009).

One way to study how the brain solves this problem is by applying temporal or spatial perturbations to learned movements, for example through delay (Vercher and Gauthier, 1992; Cunningham et al., 2001), visuomotor rotation (Cunningham, 1989), or force field (Shadmehr and Mussa-Ivaldi, 1994) paradigms. Studies have shown that we can adapt to these perturbations (for review: Shadmehr et al., 2010), but that we do not always fully update the predicted consequences of our actions (Vercher and Gauthier, 1992; Krakauer et al., 2006; Tseng et al., 2007; Galea et al., 2011; de la Malla et al., 2014; Vaswani et al., 2015). A lack of full adaptation is seen even after prolonged exposure to these perturbations (van der Kooij et al., 2016). The widely used *state space model* of adaptation (Thoroughman and Shadmehr, 2000; Donchin et al., 2003; Cheng and Sabes, 2006) captures this behavior well (Equation 1).

(1) $$x_{t}=A•x_{t-1}+B•e_{t-1}$$

The trial-by-trial adaptation is described by recursively updating the previously learned motor output *x*_*t*−1_ with a part of the error. Learning factor *B* describes how much of the error *e* is corrected on each trial. In order to account for the lack of adaptation, this model includes a retention factor *A*, which describes how much of previously learned behavior is maintained on the next trial. A multi-rate modification of this model (Smith et al., 2006), in which the motor output is the sum of a slow and a fast process, can account for features of motor learning like savings (the observation that re-adaptation is faster than the original adaptation) (Kojima et al., 2004; Ebbinghaus, 2013), anterograde interference (slower learning of an opposite perturbation) (Sing and Smith, 2010), and rapid de-adaptation/downscaling (de-adaptation is faster than the original adaptation)(Jansen-Osmann et al., 2002; Davidson and Wolpert, 2004). Unfortunately, these models are mostly descriptive in nature, and do not explain how the brain acquires its learning rate and retention factor.

**Figure 1:**
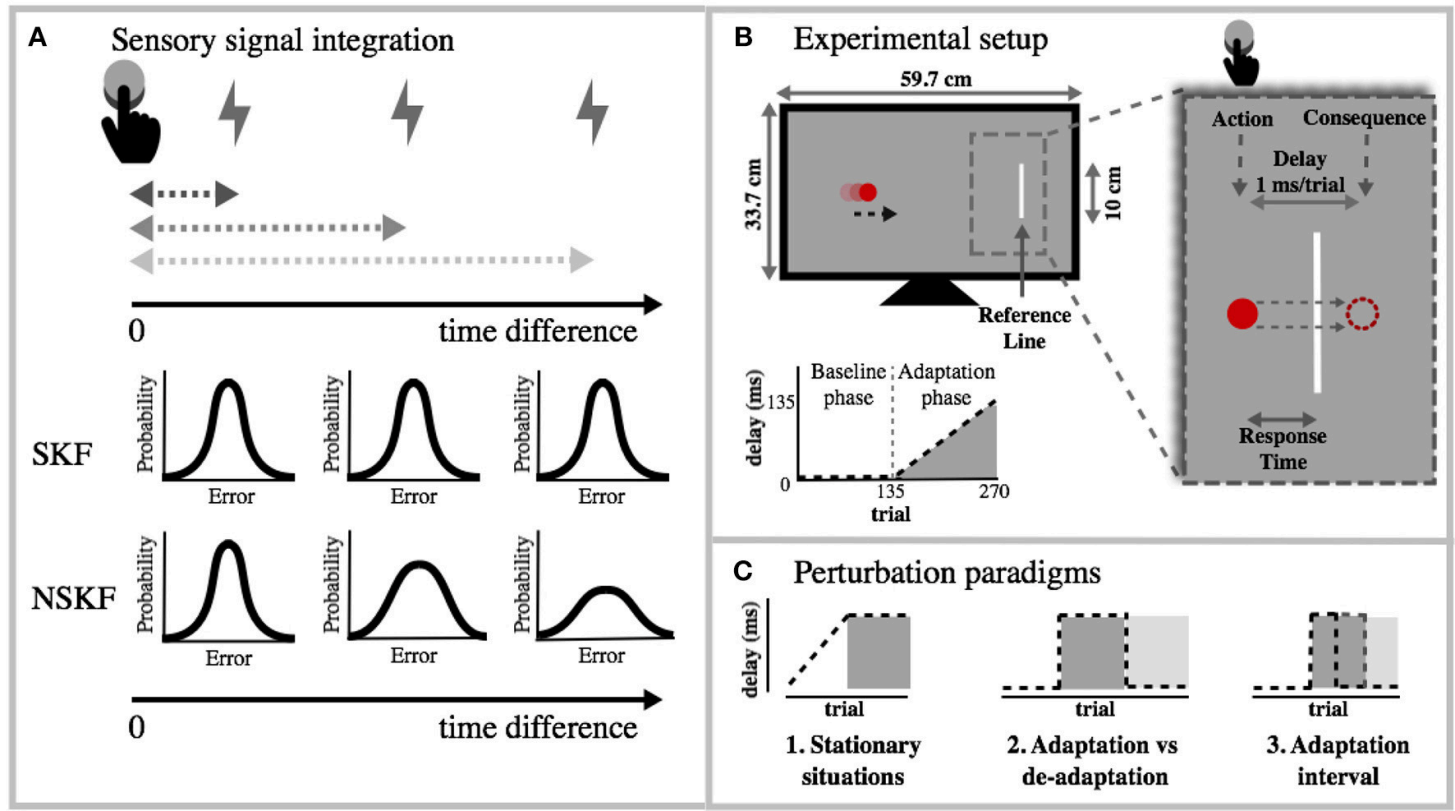
**(A)** Theoretical overview of sensory signal integration and its effect on the measurement noise. When action and consequence diverge in time, the integration becomes weaker, which in turn increases the measurement noise. This increase is the foundation for the NSKF model. **(B)** Experimental setup: Each trial, the red ball on the screen moved toward the reference line (*v* = 20, 23, or 26 cm/s; *TTC* = 1.0, 1.2, or 1.4 s). Participants were instructed to press a button (action) when the ball was as close to the vertical line as possible. The consequence of the action was delayed incrementally with 1 ms per trial. The visual consequence was the disappearance of the ball and the auditory consequence was a low pitched tone. Participants needed to press earlier to align the consequence of the action with the reference, and account for the added delay. Each condition consisted of 270 trials (of which 135 baseline trials and 135 adaptation trials). **(C)** The NSKF model was tested for different perturbation paradigms.

According to other mainstream theories of motor control, error correction can be described as an optimal process (Todorov and Jordan, 2002; Körding and Wolpert, 2004). The learning rate depends on the uncertainty of the internal model and the uncertainty of the error measurement. In support of this view it has been shown that learning decreases when the sensory feedback is noisy, whilst learning increases with larger uncertainty of feedforward estimations (Wei and Körding, 2010). In motor control, this theory has been formalized mathematically through a widely used Bayesian tool called the Kalman Filter (Kalman et al., 1960). The Kalman filter recursively updates its prediction of the future state by correcting part of the error on each trial (Korenberg and Ghahramani, 2002; Burge et al., 2008; Wei and Körding, 2010). The size of the correction depends on the reliability of the previous state estimate and the uncertainty in the new state measurement. The standard Kalman filter assumes the environment to be stationary, meaning that the process and measurement noise come from Gaussian distributions and do not change over time. The measurement noise, however, is unlikely to be stationary when temporal or spatial perturbations are applied, because these perturbations lead to dispersed feedback from different sensors. Imagine a task in which a button press causes a flash (Figure 1A). The visual timing information from the flash can be integrated with the haptic timing information from the button press based on their reliability (Ernst and Banks, 2002; Bresciani et al., 2005). Combining the sources provides more reliable timing estimations. When the flash is delayed with respect to the button press, it becomes more difficult to optimally combine the estimates from these sources of feedback (Ernst and Bülthoff, 2004; Parise et al., 2012). The larger the temporal perturbation, the more the integration is affected (Stein and Meredith, 1993). Additionally, studies have shown that estimates of time lose precision and accuracy when the temporal intervals increase (Gibbon, 1977; Wearden and Lejeune, 2008; Jazayeri and Shadlen, 2010). Consequently, the error estimates are more noisy. We therefore propose a model based on a single modification to the standard Kalman filter, in which the measurement noise can change depending on the size of the temporal perturbation.

The Kalman filter predicts the state of the world *X*_*t*_ and state-uncertainty ${\left(\sigma_{t}\right)}^{2}$ on a given trial (t) through the following set of equations (Equation 2–6):

Prediction:

(2) $$X_{t}^{-}={\hat{X}}_{t-1}$$

(3) $${\left(\sigma_{t}^{-}\right)}^{2}=\sigma_{t-1}^{2}+{\left(\sigma_{t}^{p}\right)}^{2}$$

The prediction is estimated through the posterior state-estimation ${\hat{X}}_{t-1}$ on the previous trial, and its uncertainty ${\left(\sigma_{t}^{-}\right)}^{2}$. The process variance ${\left(\sigma_{t}^{p}\right)}^{2}$ denotes the uncertainty coming from the possibility that the state of the world has changed over time.

Update:

(4) $$K\approx \frac{{\left(\sigma_{t}^{-}\right)}^{2}}{{\left(\sigma_{t}^{-}\right)}^{2}+{\left(\sigma_{t}^{m}\right)}^{2}}$$

(5) $${\hat{X}}_{t}=X_{t}^{-}+K(X_{t}^{-}-X_{t}^{m})$$

(6) $${\left(\sigma_{t}\right)}^{2}=(1-K){\left(\sigma_{t}^{-}\right)}^{2}$$

During each trial the state estimate *X*_*t*_ and the state-uncertainty estimate $\sigma_{t}^{2}$ are updated by combining the previous state prediction with the weighted prediction error of the previous trial. The prediction error is the difference between the predicted sensory feedback from the state of the world $X_{t}^{-}$ and the actual sensory feedback from the state measurement $X_{t}^{m}$. The weighing of this error is modulated by the Kalman Gain *K*. The Kalman Gain represents the reliability of the measurement ${\left(\sigma_{t}^{m}\right)}^{2}$ relative to the reliability of the state-estimate ${\left(\sigma_{t}^{-}\right)}^{2}$. These updated estimates then are used again to predict the state and state-uncertainty on the next trial.

In the standard (or stationary) Kalman filter (SKF), the measurement variance is static.

(7) $${\left(\sigma_{t}^{m}\right)}^{2}={\left(\sigma_{0}^{m}\right)}^{2}$$

However, the more our actions are temporally perturbed, the more the measurement uncertainty increases (Figure 1A). The following equation illustrates how the measurement variance is affected by the delay on each trial. Δ(σ^*m*^)^2^ denotes the change in measurement variance for each increase of the delay *D*_*t*_.

(8) $${\left(\sigma_{t}^{m}\right)}^{2}={\left(\sigma_{0}^{m}\right)}^{2}+\Delta {\left(\sigma^{m}\right)}^{2}•D_{t}$$

In this study we modified the SKF into a Non-Stationary Kalman Filter (NSKF) by replacing Equation (7) with Equation (8). This equation allows the measurement variance to increase according to the size of the delay *D*_*t*_. Larger delays will therefore lead to a smaller Kalman Gain (Equation 4), which in turn will decrease the part of the error that is corrected for.

In order to understand the behavioral patterns of adaptation when temporal perturbations are present, we test this model with unisensory and multisensory error feedback. The multisensory feedback should provide more reliable feedback estimates with regards to the unisensory feedback. We expect the measurement variance to increase when the consequence of the action is delayed in time. In addition, we expect the measurement variance to increase at a lower rate in the multisensory condition (MC) compared to the unisensory condition (UC), and consequently a higher adaptation in the MC condition than in the UC condition. Next, we simulated the results of our model for block perturbation paradigms. We expected to find a sustained lack of adaptation in stationary situations, and also expected the NSKF to be able to account for rapid de-adaptation/downscaling.

## 2. Materials & Methods

### 2.1. Participants

Ten subjects participated in the experiment. All participants gave written consent. Their vision and hearing were normal or corrected to normal. The study was part of a program that has been approved by the local ethics committee.

### 2.2. Apparatus and Stimuli

Participants were seated in front of a 27-inch led-monitor (ASUS VG278H; resolution: 1920 x 1080; refresh rate: 120 Hz; pixel size: 0.311 mm) and held a joystick (sampled at 130Hz) of which they could use either a left-handed button or right-handed button. On each trial, a vertical white *reference* line (length = 10 cm) appeared 20 cm to the right of the center of a black screen (depicted as a gray screen in Figure 1B). A solid red ball (diameter 0.6 cm) appeared, moving toward the reference line in a straight horizontal line. We used 3 different movement speeds (*v* = 20, 23, or 26 cm/s) and 3 different time to contact durations (*TTC* = 1.0, 1.2, or 1.4 s). This way, the ball could appear at 9 different distances from the reference line. The participants were instructed to press the button when the ball was as close as possible to the reference line. The feedback they received differed between conditions. In the Unisensory Consequence (UC) conditions, the ball disappeared and in the Multisensory Consequence (MC) conditions the ball disappeared and a low pitched tone was played. From the sensory consequence of the button press, participants perceived the size of their temporal error. We did not provide any further feedback about the “correctness” of the participant's responses. Each condition consisted of a baseline and a perturbation phase. Both phases consisted of 135 trials. During the baseline phase there was no perturbation. In the perturbation phase the consequence of the participants' action was delayed incrementally with 1 ms per trial, resulting in a maximal delay of 135 ms. At the end of each experiment, we asked participants if they had consciously perceived any delay or conflict in the feedback they received. If the delays were consciously perceived, this would affect the sense of agency of the consequence of the action. None of the participants reported noticing the delay in the consequence of their action.

### 2.3. Analysis of Responses

We recorded the response time (RsT), i.e., the time difference between the button press and the moment the ball reached the target line (Figure 1B). Positive RsT denoted that the action preceded the ball crossing the line. During this experiment, we delayed the consequence of the action incrementally with 1 ms/trial. In order to adapt to this increasing temporal perturbation, participants would need to initiate the action earlier with increasing delays, i.e., have more positive RsT. We analyzed individual behavior by inspecting the RsT and its simple moving average (window = 10 trials). It seemed that baseline variance was negatively affecting the adaptation. We verified this effect by calculating the Pearson correlation of the baseline variability and RsT during adaptation. Furthermore, we tested for one-sided differences in adaptation with a paired t-test between the MC and UC condition (α = 0.05).

### 2.4. Modeling

We modeled the SKF and the NSKF on the data of each participant. We fitted the models with the help of the *fkf* function from the FKF-package (Luethi et al., 2018) in the *R* program (R Core Team, 2013). This function is designed to implement the Kalman filter by iteratively predicting the next state according to Equations (1–6).

The Kalman filter was updated by introducing the temporal perturbation as a new measurement of the state $X_{t}^{m}$ on each trial. The difference between the two models comes from the expected effect of the temporal perturbation on the measurement noise (Equations 7 vs. 8). In the SKF model, Δ(σ^*m*^)^2^ was zero and therefore the (σ^*m*^)^2^ equaled ${\left(\sigma_{0}^{m}\right)}^{2}$ during the entire experiment (Equation 7). ${\left(\sigma_{0}^{m}\right)}^{2}$ was approximated by calculating the participants' baseline variance. For the NSKF model, we calculated the increase in measurement noise Δ(σ^*m*^)^2^ per 1 ms delay (Equation 8), in addition to the ${\left(\sigma_{0}^{m}\right)}^{2}$ (baseline variance). Δ(σ^*m*^)^2^, was a free parameter in the model. The Δ(σ^*m*^)^2^ that resulted in the least squared error between the prediction of the Kalman filter and the RsT data was calculated. It was calculated separately for the UC and MC condition.

The following initial parameters were used for both models: ${\hat{X}}_{0}^{-}=0$ ms, ${\left(\sigma_{0}^{-}\right)}^{2}=0.25$ ms, and ${\left(\sigma_{t}^{P}\right)}^{2}=4\cdot 10^{-6}$ ms^2^.

The output from the model provided the state prediction $X_{t}^{-}$ and the Kalman Gain *K*. We expected ${\left(\sigma_{0}^{m}\right)}^{2}$ and Δ(σ^*m*^)^2^ to be larger for the UC condition and checked for these one-sided significant differences between conditions with *t*-tests (α = 0.05). We reported bootstrapped confidence intervals (95%).

### 2.5. Other Perturbation Paradigms

**Figure 2:**
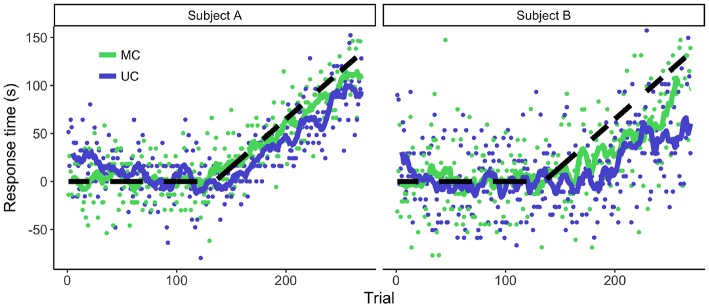
Adaptive behavior of two representative participants during the temporal perturbation experiment. Dots denote the individual responses per trial and the lines are the moving averages. Positive values denote an early response. The dashed line represents the temporal perturbation. The more the responses followed this line, the more successful the adaptation to the temporal perturbation.

**Figure 3:**
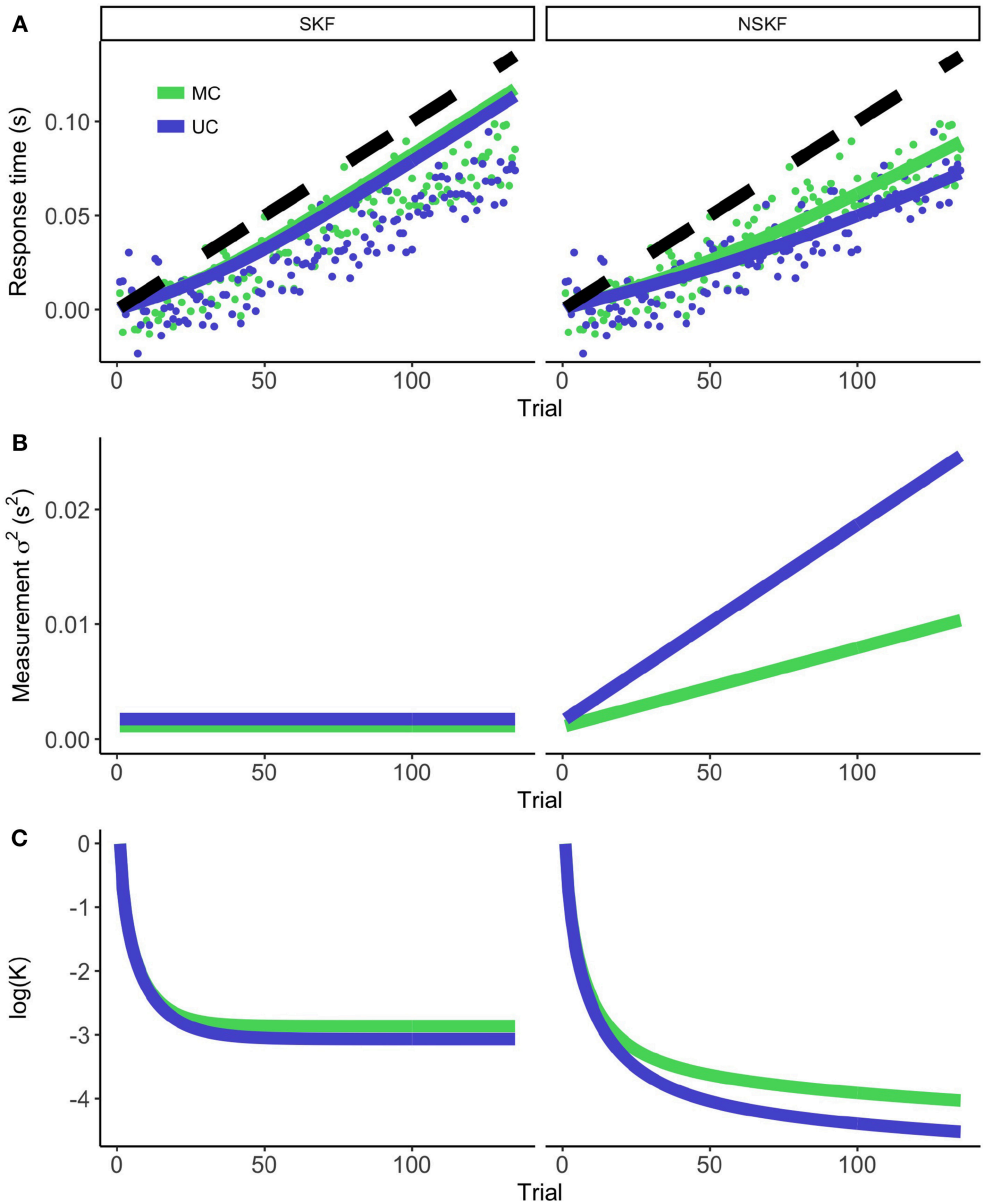
The modeling results for the stationary (left) and non-stationary (right) Kalman filter. **(A)** The average response times (dots) and model prediction (colored lines) for the MC and UC condition. The black dashed line represents the temporal perturbation of the action during the experiment. **(B)** The measurement noise as calculated from equation 7 (SKF) and 8 (NSKF). **(C)** The resulting log(Kalman Gain).

In order to see how our model would perform to other types of perturbation paradigms, we extrapolated the measurement variance (σ^*m*^)^2^ to different situations. We were interested to see how our model would behave when the perturbation was stationary (Figure 1C-1) and when the perturbation was suddenly removed (Figure 1C-2). Based on results from previous studies (Jansen-Osmann et al., 2002; Davidson and Wolpert, 2004; Smith et al., 2006), we expect faster de-adaptation compared to the initial adaptation. On top of that, we expected that longer adaptation phases would cause longer de-adaptation (Figure 1C-3). We tested these hypotheses by fitting exponential functions on the model output and calculating the time constants of these functions.

## 3. Results

### 3.1. Behavioral Analysis

We exposed participants to a temporal perturbation paradigm and analyzed the adaptive behavior. Figure 2 shows the behavior of two representative participants. The participants started accounting for the delay, meaning that they pressed the button earlier in order to decrease the temporal error. As expected, there was a lack of full adaptation to the perturbation (for all but one participant).

The degree of adaptation varied among participants. A factor that seemed to influence the amount of adaptation was the variability of the participants (Figure 2). We confirmed this observation by calculating the correlation between the baseline variance and the sum of the response times during the adaptation phase. Higher RsT variability in the baseline phase seemed to be correlated with lower average RsT during the adaptation phase [*r*(18) = –0.5, *p* = 0.02]. On average, there were more positive RsT in the MC than in the UC condition for most participants [*t*(18) = 4.8, *p* < 0.001], providing further evidence that the lack of adaptation could have to do with changes in measurement noise.

### 3.2. Increases in Measurement Noise Can Account for Lack of Adaptation

Figure 3 demonstrates the modeling results of the SKF and NSKF for the MS and US condition. The participants showed adaptive behavior by initiating the button press earlier (i.e., more positive RsT) over time. The SKF predicted an adaptation that was larger than observed in the participants, and parallel to the temporal perturbation that was introduced. As expected, the NSKF described the data more accurately, with lower weights for new measurements as the temporal perturbation grew larger. This is reflected in an increasing ${\left(\sigma_{t}^{m}\right)}^{2}$ (Figure 3B), showing that the best fit of the NSKF model is achieved with a non-stationary measurement noise. The Kalman Gain (presented in a log-scale in Figure 3C) reaches its asymptote in the SKF, while it keeps declining in the NSKF. This means that the weight on error measurements decreases as the temporal perturbation increases in the NSKF model.

### 3.3. Smaller Increase of Measurement Variance for Multisensory Condition

**Figure 4:**
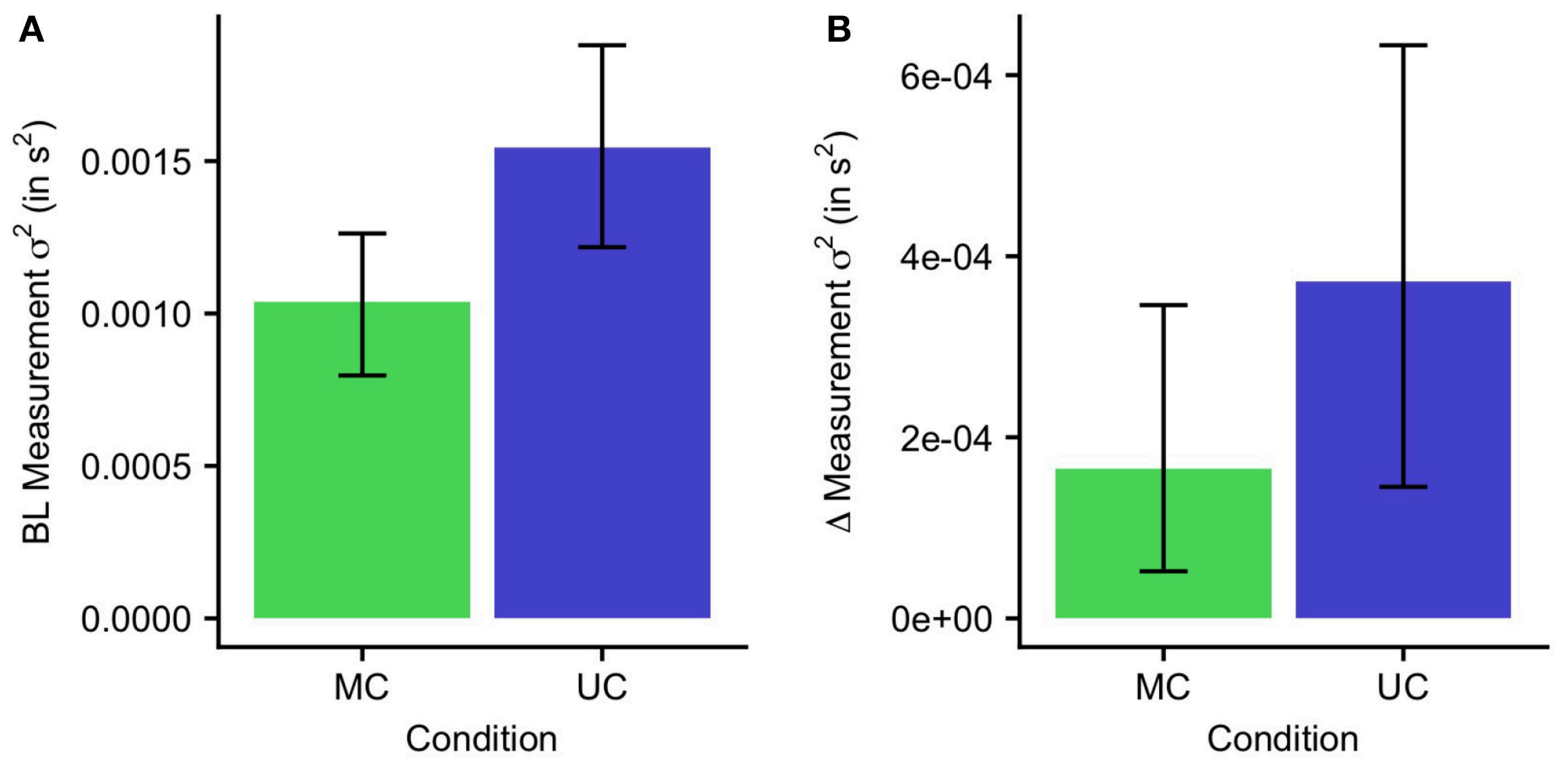
Measurement variance for the MC and UC condition. Error bars indicate 95% confidence intervals. **(A)** Baseline measurement variance. **(B)** The increase of measurement variance on each trial.

In order to quantify the effect of temporal perturbations on the measurement noise, we examined the differences between the MC and UC condition for ${\left(\sigma_{0}^{m}\right)}^{2}$ and Δ(σ^*m*^)^2^ (Figure 4). ${\left(\sigma_{0}^{m}\right)}^{2}$, approximated by the baseline RsT variance, was higher for the UC condition (0.0015 *s*^2^) than for the MC condition [0.0010 *s*^2^; *t*(9) = 4.4, *p* < 0.001]. Similarly, Δ(σ^*m*^)^2^ was higher in the UC condition (3.7 ·10^−4^ *s*^2^) than the MC condition [1.7 ·10^−4^ *s*^2^; *t*(9) = 1.9, *p* = 0.04]. The lower measurement variance in the multisensory compared to the unisensory condition led to improved learning and more complete adaptation.

### 3.4. Accounting for Lack of Adaptation to Stationary Situations and Rapid De-adaptation

**Figure 5:**
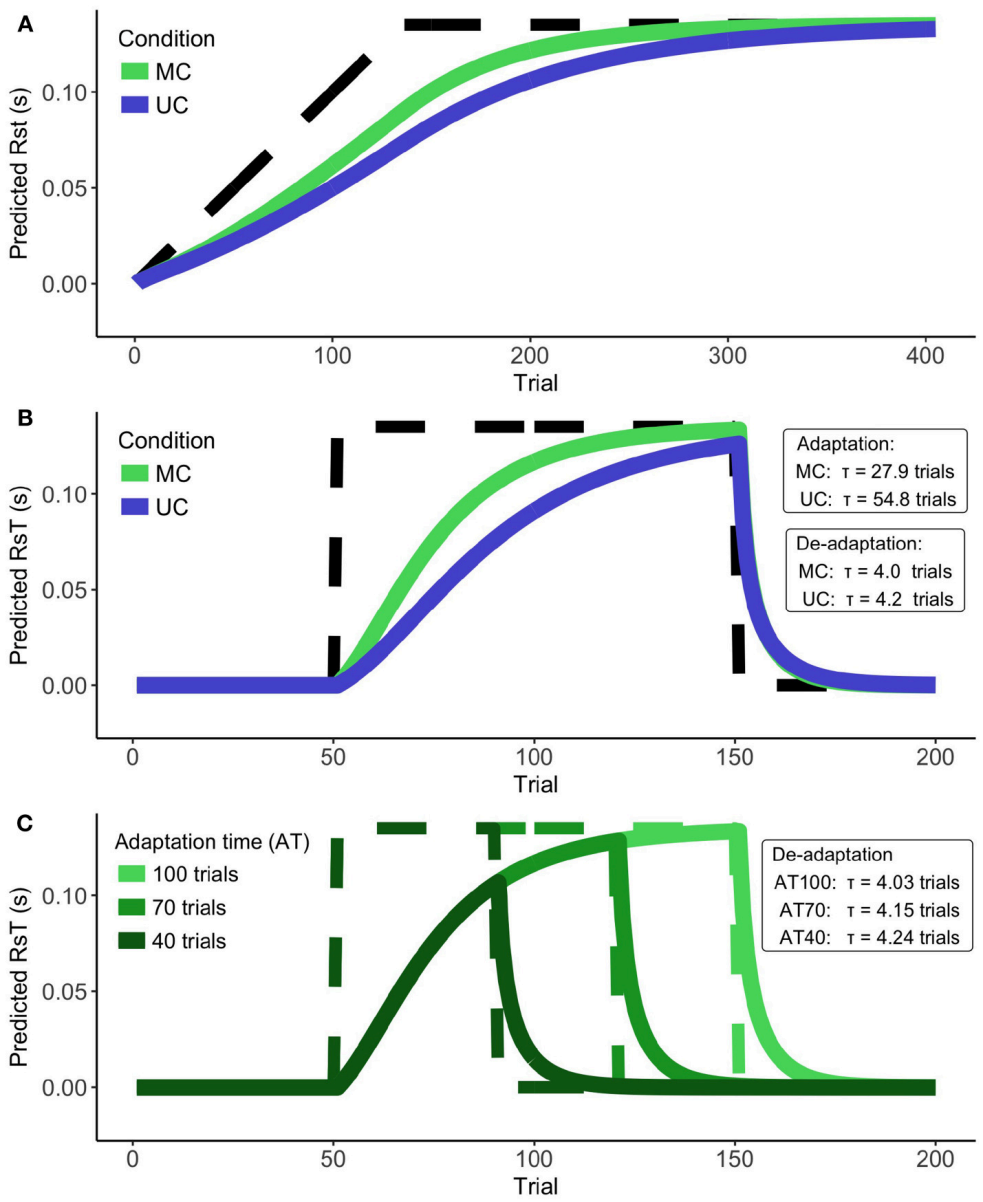
Model predictions. **(A)** The conversion of the Kalman filter during stationary perturbations for Unisensory and Multisensory feedback. Perturbation denoted by dashed line. **(B)** Speed of adaptation vs. de-adaptation for Unisensory and Multisensory feedback. Perturbation denoted by dashed line. **(C)** Speed of de-adaptation after different lengths of the adaptation phase. Perturbation denoted by dashed lines.

We applied our model to a number of different perturbation paradigms (Figure 1). Figure 5A shows predictions for an adaptation to stationary perturbations (i.e., where the delay is fixed to 135 ms). We can see that the NSKF predicts the participants' behavior to converge with the input after about 250–300 trials. It therefore does not predict the lack of adaptation to stationary perturbation paradigms as described in the literature.

As expected, we found a much faster de-adaptation compared to the initial predicted adaptation (Figure 5B). This predicted de-adaptation was slightly faster for the MC- than the UC condition. When we examine the effect of the length of the adaptation phase on the speed of de-adaptation for the MC condition (Figure 5C), we find de-adaptation to be faster for longer adaptation phases. This finding was opposite to the results reported in the literature. Increasing de-adaptation times can thus not be explained by the NSKF.

## 4. Discussion

In this study, we aimed to provide a possible explanation for the decreasing amount of error correction to gradually increasing temporal perturbations. We have shown that increasingly noisy feedback estimates that arise from accumulating delays can explain the pattern of adaptation observed in our participants. When feedback is delayed in time, its uncertainty increases. The coupling between the participant's action and its consequence becomes more noisy due to diverging sensory signals, impeding sensory integration (Stein and Meredith, 1993; Ernst and Bülthoff, 2004; Parise et al., 2012).

We are not the first to demonstrate that noise affects the amount of learning from an error. The amount of learning from an error has been shown to increase when state estimates are more uncertain, while learning rates decreased when measurement estimates are more uncertain (Wei and Körding, 2010). The Kalman filter has previously been used to show that the measurement uncertainty could effect the amount of adaptation to a perturbation (Burge et al., 2008; Haith et al., 2008). Furthermore, recent studies have provided evidence that multisensory feedback can benefit performance in a delay detection task (van Kemenade et al., 2016, 2017; Straube et al., 2017). However, to our knowledge we are the first ones to model the behavioral patterns during adaptation to incremental temporal delays by increasing the measurement variance. The model successfully predicted lower weights for new measurements when temporal perturbations became larger, as was observed in the behavioral data.

We chose to apply gradually increasing temporal delays in order to verify the NSKF model. The first reason for this approach was that the gradual delays simulated the effect of small changes that occur in muscles during exercise. Fatigue and temperature changes for example, are thought to increase the electromechanical delays in muscles over time (Zhou et al., 1998). Secondly, it gave us the opportunity to estimate the increment of measurement noise with the increasing delay. A sudden stationary perturbation could have also served to calculate increases in measurement noise, but it may have affected other factors in the model, like the process variance (Narain et al., 2013), or the sense of agency with regards to the sensory consequence of the action (Rohde et al., 2014; Rohde and Ernst, 2016).

In order to decrease the amount of free parameters in the model, we used baseline RsT-variability to reflect the measurement variance of the model in the unperturbed state. These variabilities are correlated, but the RsT variability likely overestimates the actual baseline measurement noise in the system.The reason being that motor variability includes, along with measurement noise, planning noise (Churchland et al., 2006a,b; van Beers, 2009), execution noise from the motor neurons (Harris and Wolpert, 1998; Jones et al., 2002; van Beers et al., 2004; Faisal et al., 2008), noise from imprecise estimates of the speed of the target (Osborne et al., 2005) and perturbations from the environment (Tan et al., 2016). However, any differences between the UC and MC condition are expected to be due to differences in measurement noise. By using the baseline variability, we filter out any baseline differences between the conditions. As a result, we can assume that differences in (Δσ^*m*^)^2^ stem from the temporal perturbation. Even though this might underestimate the size of (Δσ^*m*^)^2^, it does preserve the trend.

The multi-rate state space model of Smith et al. (2006) is a simple descriptive model that can account for a series of motor learning features, like savings (Kojima et al., 2004; Ebbinghaus, 2013), anterograde interference (Sing and Smith, 2010), lack of adaptation (Krakauer et al., 2006; Tseng et al., 2007; Galea et al., 2011; de la Malla et al., 2014; Vaswani et al., 2015), and fast de-adaptation (Jansen-Osmann et al., 2002; Davidson and Wolpert, 2004). It has been proposed that savings and anterograde interference are features of motor learning that are expressed in the final adaptation. However, it is more likely that these features stem from different *model free* types of learning processes: use-dependent plasticity and operant reinforcement (Huang et al., 2011). We believe that at least some of the other proposed features stem from changes in measurement noise due to temporal perturbations. The NSKF accounts for decreased sensorimotor learning due to perturbation-induced measurement noise. One disadvantage of the Kalman filter is that the predicted state, with stationary perturbations, converges to the input (Burge et al., 2008). This is even the case with increased measurement noise, as the Kalman gain never completely reaches zero. In reality however, people do not fully adapt to the perturbation, even after prolonged exposure (van der Kooij et al., 2016). The persisting adaptation bias can therefore not be explained by our single modification of the standard Kalman filter. Furthermore, the model predicts fast de-adaptation, which is consistent with reported behavior in other studies (Jansen-Osmann et al., 2002; Davidson and Wolpert, 2004; Smith et al., 2006). The multi-rate model predicts a decreasing speed of de-adaptation with longer adaptation phases (Smith et al., 2006), while our model predicts the opposite effect: longer adaptation times predicted slightly smaller time constants for de-adaptation. However, this feature of motor learning could justifiably be attributed to changing uncertainties of the state-estimate (as modeled by Narain et al., 2013), rather than measurement uncertainty. Unexpected perturbations in the environment increase the uncertainty of the state-estimate (Tan et al., 2016). As a result, the Kalman Gain temporarily increases as well. Over time, the Kalman Gain decreases again. The sooner de-adaptation happens after the original adaptation, the higher the Kalman gain. Though in theory, this idea could explain how de-adaptation becomes slower over time, more research is needed on how sudden perturbation affect the Kalman Gain. We decided against modeling the process variance as a non-stationary parameter, due to its implications on the interpretation of the measurement variance. Future models could aim to explore these relationships further.

Even though our model does not account for lack of adaptation in stationary situations, higher levels of measurement noise do likely have an effect, even over longer periods of time. It has been recently shown that people can correct for errors near-optimally over time, but not from trial to trial (van Beers et al., 2013). In order to be optimal, the brain needs to distinguish a bias in prediction errors from other sources of noise (Rohde and Ernst, 2016). Through experience, the brain can learn from signals and become more optimal (van Beers et al., 2013). The noisier the signal, the more experience is required. It is possible that, at a certain noise level, the time to reach the required experience becomes infinite. If this were the case, prediction errors would be perceived as noise in the system and adaptation would stall. This would also explain why more variable participants of this study were likely to adapt less than participants with a lower variability.

Additionally it has been shown that the learning rate during prism adaptation (Kitazawa et al., 1995; Tanaka et al., 2011) and visuomotor rotation adaptation (Honda et al., 2012) is affected by delayed temporal feedback. This finding provides further evidence that measurement noise is higher when sensory feedback is delayed. Interestingly, Honda et al. (2012) found that the adverse effect of the delay on the learning rate was alleviated when adaptation to the delay took place before the adaptation to the visuomotor rotation. This might be due to a possible effect of causal binding on the measurement noise. Causal binding refers to the observation that delays between an action and its sensory consequence can cause a recalibration of the perceived timing of the consequence (Stetson et al., 2006; Buehner, 2012). When exposed to a delay, the temporally conflicting signals from action and consequence start to be perceived as more simultaneous. Removing the delay causes an after-affect in which the previously delayed sensory consequence is perceived to lead the timing of the action. The brain constantly needs to estimate the uncertainty of different signals (Knill and Pouget, 2004). As causal binding is responsible for decreasing the perceived size of the sensory conflict, it could have a similar effect on the estimated measurement uncertainty. Conversely, Tanaka et al. (2011) did not find an increased learning rate after an adaptation period, even though the subjective delay was decreased. The main difference between the studies of Tanaka et al. (2011) and Honda et al. (2012) is the type of feedback provided (final movement error and continuous movement error respectively). It has been previously shown that the type of feedback provided affects the adaptation (de la Malla et al., 2012). Final movement feedback provides a lower error signal-to-noise ratio, regardless of the delay. The decrease of the perceived measurement noise due to causal binding might be minimal in more noisy signals. Similarly, studies in delay detection have shown experiments that only provide final movement error show larger benefits of multimodel feedback than studies that provide continuous movement feedback (van Kemenade et al., 2016). These differences might also be attributed to signal-to-noise differences, giving a higher benefit of multisensory feedback in tasks with more noisy feedback types.

The present study explored possible adaptation differences due to discrepancies in uncertainty between unisensory and multisensory delayed feedback. The cerebellum has generally been proposed as the brain area that is involved in computing the internal forward model (Miall et al., 1993; Wolpert and Miall, 1996; Wolpert et al., 1998; Bastian, 2006; Tseng et al., 2007), comparing forward predictions with sensory feedback (Blakemore et al., 1998, 1999; van Kemenade et al., 2018). A recent study on unisensory vs multisensory differences in a delay detection task suggests that the angular and middle temporal gyrus are involved in detecting cross-sensory prediction errors (van Kemenade et al., 2018). These areas, connected to the cerebellum, are a likely candidate for the benefit of multisensory over unisensory temporal signals, though further examination is desirable. Further research into the effects of unisensory and multisensory feedback on brain processes will benefit our knowledge on how prediction errors are processed and how the brain deals with sensory noise during adaptation.

A limitation of our study is the absence of a unisensory auditory condition. The inclusion of this condition would have shed some light on how noise in auditory feedback is affected by delays. However, previous research has shown that multisensory feedback affects the reliability of the estimates (Ernst and Banks, 2002; Bresciani et al., 2005), and therefore we do not expect any effect of this limitation on the interpretation of our study.

In this study we focused mainly on the effect of *temporal* perturbations on the estimated measurement noise. However, an effect of spatial perturbations on the estimated measurement noise can be expected as well. Spatial perturbations often create discrepancies between different types of sensory information, impeding integration. Our study shows that these perturbation paradigms we use to study motor learning lead to increased measurement noise. It is important to keep this in mind when interpreting the results of perturbed actions during a sensorimotor tasks. Most importantly, we have shown these increases in measurement noise can account for a lack of adaptation to perturbation paradigms.

## Data Availability

Data is freely available at https://osf.io/tjkby/.

## Author Contributions

EK and JL-M contributed to the conception of the work, data analysis, and results interpretation. EK wrote the manuscript and both authors contributed to the revision and approval of the submitted version.

### Conflict of Interest Statement

The authors declare that the research was conducted in the absence of any commercial or financial relationships that could be construed as a potential conflict of interest.
